# Cost Savings and Efficacy in Management of Paracetamol Poisoning in a 23-hours Emergency Department Observation Unit: A Comparison to Inpatient Care

**DOI:** 10.7759/cureus.6294

**Published:** 2019-12-05

**Authors:** Kelvin K Kuan, Hoon Chin Lim, Geraldine Goh, Gabriel S Arciaga, Pak Liang Goh, Rupeng Mong, Wai Leng Chow, Hock Heng Tan

**Affiliations:** 1 Accident and Emergency, Changi General Hospital / Singhealth, Singapore, SGP; 2 Health Sciences Research, Changi General Hospital / Singhealth, Singapore, SGP

**Keywords:** acetaminophen, paracetamol, poisoning, length of stay, cost-savings, n-acetylcysteine, hepatotoxicity, observation ward

## Abstract

Introduction

Emergency department observation units (EDOU) have been shown to be effective in decreasing hospitalization rates and length of stay (LOS) for various conditions. However, cost savings and efficacy in the management of poisoning in EDOU have not been widely studied. The objective of our study is to compare the costs and effectiveness of managing paracetamol poisoned patients in the EDOU with those treated in the inpatient wards.

Methods

We conducted a historical controlled observational study comparing paracetamol-poisoned patients (who received at least 21 hours of IV N-acetylcysteine [NAC]) admitted to the EDOU during 2013-2014 with similar patients admitted to inpatient ward during 2011, 2013-2014.

Results

We found 136 patients admitted to the inpatient ward and 95 to our EDOU due to paracetamol poisoning but only 78 and 39 patients respectively fulfilled the inclusion criteria. Between the EDOU and inpatient ward groups, we found similar demographics, poisoning presentation, treatment, and adverse event profiles. There were no fatalities and only two patients (one from each group) developed hepatotoxicity. The "medical" length of stay was 31.9 hours shorter in the EDOU group compared to the inpatient ward group (23.3 versus 55.2 hours). EDOU patients have statistically significant savings (comparing bill size) of S$784 per patient.

Conclusions

Admission to the EDOU resulted in significant cost savings and 58% decreased LOS when compared to inpatient wards. The EDOU is a cost-effective and safe alternative for the management of selected paracetamol poisonings requiring NAC. Further studies would be needed to verify these results.

## Introduction

Paracetamol poisoning is the most common pharmaceutical poisoning in developed countries including Singapore [1-5]. N-acetylcysteine (NAC) is an effective antidote. A standard intravenous (IV) dose of NAC (300mg/kg) is given over 21 hours, usually requiring admission to the hospital inpatient ward. In one paper, the admission rate from the Emergency Department (ED) for paracetamol poisoning was approximately 45% for an average duration of 3.1 days [2]. Locally, admission duration was comparable at three days [6, 7].

Since its inception, observation units have been shown to be effective for the management of mild to moderate poisoning [8]. Protocol-driven emergency department observation units (EDOU) generally have a limited observation period of 24 hours, presenting a challenge to the administration of NAC [9]. However, studies have shown that management of paracetamol poisonings in an EDOU by a multidisciplinary protocol can help cut down hospital length of stay (LOS) [7, 10]. Although it is acknowledged that EDOU helps to decrease LOS and cost, there are no comparative studies on cost savings from the management of poisoning cases in EDOU versus inpatient admission [7, 9].

Our hospital EDOU protocol for poisoning commenced in end-2011 [11]. Thereafter, paracetamol-poisoned cases requiring NAC may be managed in the EDOU or the inpatient wards. We conducted a study to compare the effectiveness, safety, and costs of managing these patients who were admitted to the EDOU with those admitted to the inpatient wards. 

## Materials and methods

Study setting and population

Changi General Hospital is one of six public tertiary teaching hospitals in Singapore, with an annual estimated emergency department (ED) attendance of 150,000, representing about 15% of all ED attendances amongst the nation’s public hospitals [12]. 

NAC is started for paracetamol-poisoned patients with the possible risk of hepatotoxicity based on standard recommendations for both acute and repeated supratherapeutic ingestions (RSTI) [13]. Occasionally, empirically started NAC is stopped when subsequent assessment found it unnecessary. 

Our EDOU poisoning protocols admits patients 12 years or older, who have features of poisoning that require treatment or extended observation and are deemed likely to improve within 24 hours based on the type of poisoning and predicted clinical course [11]. Patients who do not fulfill EDOU criteria, or present with unstable vital signs, any evidence of end-organ injury or concurrent medical issues deemed to require inpatient management are excluded. Our EDOU had nine beds, managed by an emergency physician (EP) and the team in rotating shifts with support from the toxicology service. 

EPs decide on the disposition of patients presenting with paracetamol poisoning. A proportion of patients who fulfill the admission criteria to EDOU may get admitted to the inpatient ward instead. The reasons may include EP’s unfamiliarity with the EDOU protocol, lack of EDOU beds and patient preference.

Management in the EDOU aims to complete clinical care and formulate a psychosocial care plan within 23 hours. Standard NAC therapy is given as required for paracetamol poisoning. Patients may be transferred to the inpatient medical ward if they require extended NAC therapy or further evaluation of other medical conditions. Following an initial assessment by psychiatry doctors in the EDOU, patients may be discharged with an outpatient psychiatry appointment or transferred to an inpatient psychiatric service. This inpatient psychiatric service could be at our hospital (hospital psychiatric facility) or at a nearby mental health institution (external psychiatric facility). Patients admitted to the inpatient medical wards are treated for their poisoning as above and undergo similar psychiatric evaluation and disposition as needed. 

Study design and analysis

This is a historical controlled observational study of paracetamol poisoned patients presenting to our ED who were treated with at least a standard course of NAC either in the inpatient medical ward or EDOU. 

Case Selection

All patients admitted to the hospital under the International Classification of Diseases, Ninth Revision, Clinical Modification (ICD-9-CM) code of 965 (poisoning by analgesics, antipyretics and antirheumatics) were obtained from computer records from January to December of 2011, 2013 and 2014. EDOU cases admitted under poisoning protocols in 2013 and 2014 were reviewed. Cases that did not involve paracetamol poisoning were excluded. We did not include 2012 as the EDOU had just started and the number of cases admitted there was low. 

Cases selected for comparison: 1. received at least a standard 300mg/kg dose of IV NAC given over 21 hours for paracetamol poisoning and 2. met the criteria for EDOU admission. All inpatient ward admissions were reviewed by two study authors for consensus for the latter criteria. This allowed us to compare paracetamol poisoning cases of similar severity by excluding patients who are too ill for EDOU and patients who did not require NAC. 

Patients with mildly elevated initial aminotransferase (AT) [either alanine aminotransferase (ALT) or aspartate aminotransferase (AST)] levels less than two times the upper limit of normal (ULN) were included in our study. This reflects the real-world practice at our institution, where we admit such patients to the EDOU. The mildly elevated AT may be due to conditions other than paracetamol poisoning [14, 15]. The transaminases of these patients were trended during the EDOU stay. Acute liver injury is defined as AT>150 IU/L and hepatotoxicity is defined as AT>1000 IU/L [16]. 

Group Comparison

Patients admitted to the EDOU in 2013 and 2014 were compared to those admitted to inpatient wards in 2011, 2013 and 2014. A comparative analysis was performed on patient demographics, outcome, adverse events, and hospital resource use, namely LOS and estimated costs. 

We grouped selected cases into four groups for comparison: 1. pre-EDOU inpatient ward group (inpatient admissions in 2011); 2. post-EDOU inpatient ward group (inpatient admissions in 2013 and 2014); 3. all inpatient ward group (the combination of both groups 1 and 2) termed as a "ward" group; 4. EDOU group. Statistical comparisons were made between groups 2 and 4 and between groups 3 and 4. 

“Medical” LOS by hours is defined as the length of hospital stay from the time of admission to the time of hospital discharge, excluding time spent in any inpatient psychiatric facility. This does not include the time spent in the ED. This allowed us to compare the LOS required solely for the medical management of the patients. Patients in the EDOU group who were transferred to an inpatient medical ward for further management were analyzed with the EDOU group.

All data was entered directly into a standardized Microsoft Excel (Microsoft, Redmond, Washington, US) spreadsheet. A descriptive analysis was performed. Categorical data are presented as percentage frequencies, while continuous data are presented as medians with inter-quartile ranges (IQR). Statistical analyses were performed with data analytic software on GraphPad (GSL Biotech LLC, Chicago, US). The level for statistical significance was set at p<0.05. 

Financial Analysis

To calculate the financial cost of the hospital visit, we could not exclude the LOS when the patient was transferred under our hospital psychiatric facility. This is because the extraction of cost is based on the whole LOS duration in our hospital and hence, we reported it as "actual LOS" for the whole hospital stay, with the costs involved. This cost only includes expenses within the hospital stay and ends when patients were discharged or transferred out of the hospital. All costs are presented in Singapore dollars (S$).

The primary cost outcome was the bill size difference between the two cohorts. To compare across the years, the quantity for each service code was inflation-adjusted using a price list generated in March 2018 for each patient. A generalized linear model using the gamma family and log link was used to predict costs with cohort and LOS as independent variables. The choice of the gamma distribution was confirmed by the modified Park test. Several goodness-of-fit tests (Pearson correlation, Pregibon’s link and the modified Hosmer-Lemeshow tests had insignificant p-values) indicated the acceptability of the log-link function. Using recycled predictions, the cost difference between the cohorts was computed assuming the same covariate balance in both groups. As the cost and LOS data was skewed, bootstrapped 95% confidence intervals (CIs) around the means are reported. 

## Results

**Figure 1:**
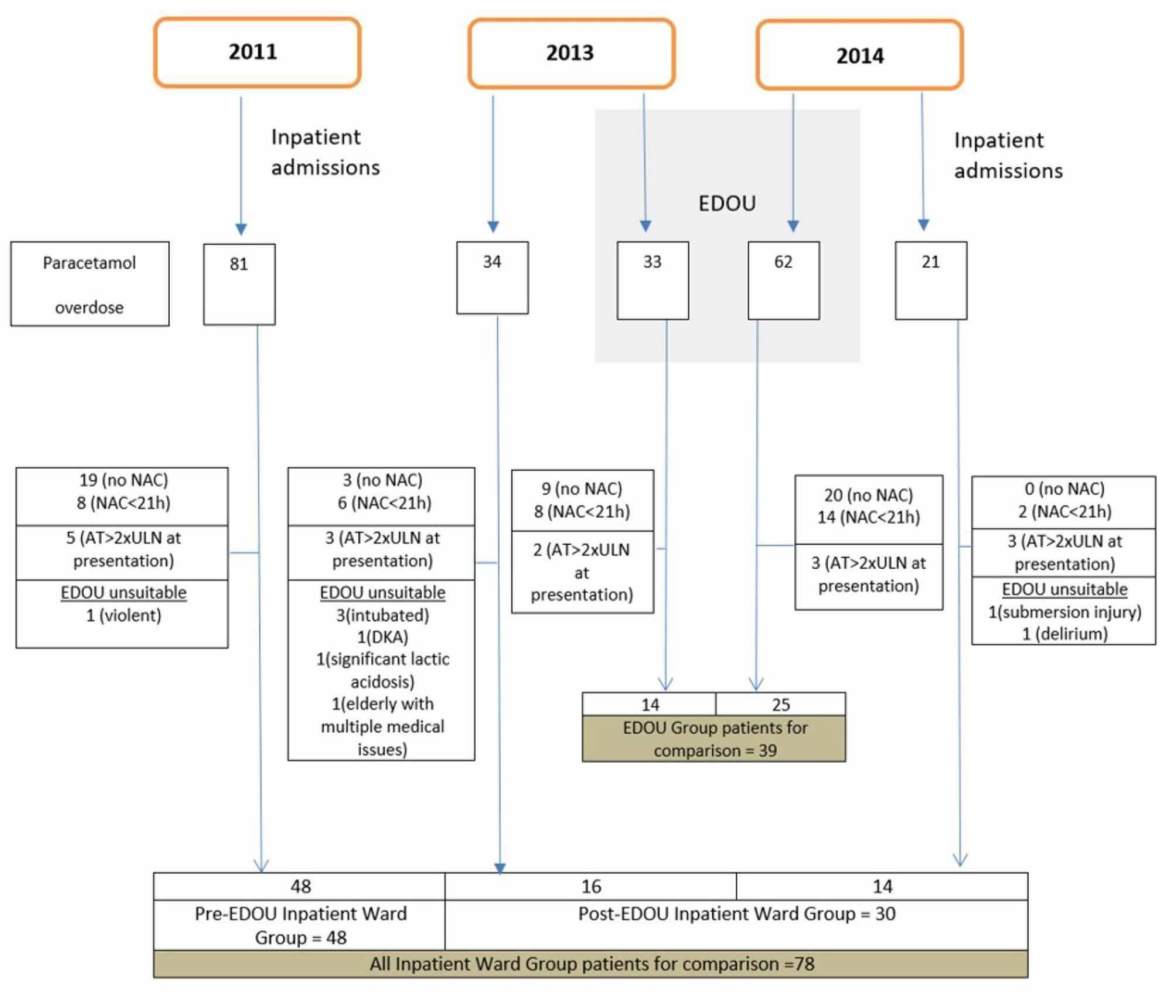
Patient inclusion and exclusion NAC - N-acetylcysteine; AT - aminotransferase; ULN - upper limit of normal; EDOU - emergency department observation units

Of the 153 ward admissions (91 cases in 2011, 36 in 2013 and 26 in 2014) identified under ICD-9-CM code 965 in the study period, 136 involved paracetamol poisoning. We found 220 EDOU admissions (66 cases in 2013 and 154 cases in 2014) under the poisoning protocol, of which 95 involved paracetamol poisoning. We used 78 cases ("ward" group) and 39 cases (EDOU group) for comparison. We excluded 58 patients from the "ward" group and 56 patients from the EDOU group. The reasons for their exclusion are found in Figure 1 below. 

Table 1 describes the demographics and characteristics of these patients. Comparison of the different groups (2 versus 4 and 3 versus 4) showed that they were similar in age and distribution, with no statistical differences in gender, race and presenting symptoms.

**Table 1:** Patient characteristics and results NC-not calculated; NAC - N-acetylcysteine; EDOU - emergency department observation units; g - grams Statistical tests: ^a ^2-tailed Mann-Whitley U test; ^b^ Fisher's exact test; ^c ^Chi-Square test ^d ^Others: include unknown time of ingestion and RSTI cases

Patient groups		Group 1 : pre-EDOU inpatient ward group (2011) (n=48)	Group 2: post-EDOU inpatient ward group (2013, 2014) (n=30)	Group 3: all inpatient ward group (2011, 2013, 2014) (n=78)	Group 4: EDOU group (2013, 2014) (n=39)	P value: Groupp 2 vs 4 (post-EDOU inpatient ward group vs EDOU group)	P value: Group 3 vs 4 (all inpatient ward group vs EDOU group)
Median age (years)^a ^		24 (19.8-32.3)	28.5 (21.3-36.8)	25.5 (20-34.8)	24 (19-35)	0.267	0.509
Gender (%)^b^	Male	10 (20.8)	11 (36.7)	21 (26.9)	7 (17.9)	0.101	0.361
	Female	38 (79.2)	19 (63.3)	57 (73.1)	32 (82.1)		
Race (%)^c^	Chinese	31 (64.6)	20 (66.7)	51 (65.4)	26 (66.7)	0.715	0.861
	Malay	7 (14.6)	4 (13.3)	11 (14.1)	6 (15.4)		
	Indian	6 (12.5)	3 (10)	9 (11.5)	5 (12.8)		
	Others	4 (8.33)	3 (10)	7 (8.97)	2 (5.13)		
Past medical history (%)^b^	Alcoholism	1 (2.08)	2 (6.67)	3 (3.85)	0 (0)	0.185	0.550
	Liver cirrhosis	0 (0)	0 (0)	0 (0)	0 (0)	1	1
Type (%)^b^	Acute	46 (95.8)	29 (96.7)	75 (96.2)	38 (97.4)	1	1
	Repeated supratherapeutic ingestion (RSTI)	2 (4.17)	1 (3.33)	3 (3.85)	1 (2.56)		
Intent (%)^b^	Deliberate self-harm	39 (81.3)	30 (100)	69 (88.5)	36 (92.3)	0.252	0.748
	Accidental	9 (18.8)	0 (0)	9 (11.5)	3 (7.69)		
Co-ingestants (%)^b^	Alcohol	9 (18.8)	3 (10)	12 (15.4)	2 (5.13)	0.646	0.137
	Analgesics	3 (6.3)	3 (10)	6 (7.70)	2 (5.13)	0.646	0.717
	Antihistamines	3 (6.3)	4 (13.3)	7 (8.97)	2 (5.13)	0.392	0.716
	Antidepressants	0 (0)	1 (3.33)	1 (1.28)	1 (2.56)	1	1
	Benzodiazepines	2 (4.17)	2 (6.67)	4 (5.13)	0 (0)	0.185	0.300
	Caffeine	9 (18.8)	3 (10)	12 (15.4)	5 (12.8)	1	0.788
	Orphenadrine	4 (8.33)	6 (20)	10 (12.8)	3 (7.69)	0.163	0.539
	Others	9 (18.8)	1 (13.3)	13 (16.7)	5 (12.8)	0.223	0.787
	None	20 (41.7)	13 (43.3)	33 (42.3)	23 (59.0)	0.231	0.117
Symptoms (%)	Vomiting	28 (58.3)	8 (26.7)	36 (46.2)	16 (41.0)	NC	NC
	Abdominal pain	16 (33.3)	8 (26.7)	24 (30.8)	11 (28.2)		
Aminotransferase (AT) on presentation (%)^b^	Normal	40 (83.3)	28 (93.3)	68 (87.2)	36 (92.3)	1	0.540
	Abnormal (AT>1x, AT<2x)	8 (16.7)	2 (6.67)	10 (12.8)	3 (7.69)		
Highest aminotransferase level (IU/L)^c^	AT <150	48 (100)	27 (90.0)	75 (96.2)	38 (97.4)	0.233	0.471
	150< AT<1000	0 (0)	2 (6.67)	2 (2.56)	0 (0)		
	AT>1000	0 (0)	1 (3.33)	1 (1.28)	1 (2.56)		
Dose ingested (g) median (interquartile range)^a^		10 (9-16.0)	11 (9-18)	10 (9-17.3)	12 (10-15)	0.902	0.641
Decontamination (%)^b^	None	37 (77.1)	27 (90)	64 (82.1)	32 (82.1)	0.496	1
	Activated charcoal	11 (22.9)	3 (10)	14 (17.9)	7 (17.9)		
NAC duration (%)^b^	20-21 hours	43 (89.6)	27 (90)	70 (89.7)	36 (92.3)	1	0.750
	>21 hours	5 (10.4)	3 (10)	8 (10.3)	3 (7.69)		
NAC initiation post-ingestion (%)^b^	<8 hours	31 (64.6)	19 (63.3)	50 (64.1)	27 (69.2)	0.777	0.513
	>8 hours	15 (31.25)	8 (26.7)	23 (29.5)	9 (23.1)		
	Others^d^	2 (4.2)	3 (10)	5 (6.4)	3 (7.7)	NA	NA
Anaphylactoid reactions (%)^b^		5 (10.4)	0 (0)	5 (6.41)	2 (5.13)	0.505	1
Psychiatry review (%)^b^		41 (85.4)	29 (96.7)	70 (89.7)	35 (89.7)	0.379	1

We note that the majority of our patients ingested paracetamol for reasons of deliberate self-harm (81.3-100%). For the comorbidities studied in our cohort, we found only three cases of known alcohol dependence in the "ward" group. About half of the patients in both groups took co-ingestants, with similar rates of common co-ingestants like alcohol, caffeine, and orphenadrine. There was no significant difference in the median paracetamol dose ingested or the time of NAC initiation post-ingestion.

Four patients had liver injury with peak AT > 150 IU/L (described in Table 2). Two of the patients (patients 1 and 2) had initial AT that is slightly elevated but <2x ULN. The other two patients developed hepatotoxicity (patients 3 and 4). All the patients recovered well. There were no cases of liver failure requiring a transplant and there were no fatalities. 

**Table 2:** Patients with acute liver injury and hepatotoxicity *Elevated ALT or AST; EDOU - emergency department observation units; ALT -  alanine aminotransferase; AST - aspartate aminotransferase; AT - aminotransferase; NAC - N-acetylcysteine; LOS - length of stay

No.	Year	ALT/AST (AT) (IU/L) on presentation	Highest AT (IU/L)	Paracetamol dose i ingested (g)	NAC initiation from ingestion < 8 hours	NAC duration (hours)	"Medical" LOS (hours)
1	2011 ward	70*/38	174	16	Yes	150	178
2	2014 ward	58*/60*	469	20	No	45	129
3	2014 ward	18/26	1561	51.4	Yes	61	180
4	2013 EDOU	9/23	1894	Unknown	Yes	33	133

Table 3 describes the outcome of the patients after admission to inpatient wards and EDOU. Five patients (12.8%) were transferred from EDOU to the inpatient medical ward. Of these, two had extended NAC treatment, one for international normalized ratio (INR) of 1.4 without transaminitis and the other is described in table 2 (patient 4). The other three patients were admitted for (i) altered mental state secondary to orphenadrine, (ii) initial refusal for NAC in EDOU and (iii) right hypochondrial pain and raised INR without transaminitis respectively. "Medical" length of stay in the "ward" group was 55.2 hours, significantly longer by 31.9 hours compared to that in the EDOU group at 23.4 hours (p<0.0001). A subgroup analysis excluding patients with AT>150 IU/L did not show any difference for LOS (Table 3). 

**Table 3:** Patient discharge outcomes LOS - length of stay; EDOU - emergency department observation units ^ a^ from time of admission to discharge or transfer to a psychiatric service (in our hospital or external facility)

	Pre-EDOU inpatient ward group	Post-EDOU inpatient ward group	"Ward" group	EDOU group
Cases	48	30	78	39
Discharged	42	23	65	23
Discharged against advice	1	2	3	2
Transferred to inpatient medical ward	NA	NA	NA	5
Transferred to hospital psychiatric facility	4	2	6	3
Transferred to external psychiatric facility	1	3	4	6
"Medical" LOS (hours) median (interquartile range)^a^	55.2 (42.2 -61.6)	54.5 (39.5-63.0)	55.2 (39.6-62.6)	23.4 (22.4-24.0)
"Medical" LOS (hours.) median (interquartile range); excluding liver injury (n = 4)	54.8 (41.3-61.0); (n = 47)	51.6 (39.2-62.1); (n = 28)	54.0 (39.5-61.6); (n = 75)	23.4 (22.4-23.9), (n = 38)

Financial impact results

Table 4 describes the average LOS (in days) and cost outcomes for the "ward" group and the EDOU group. We found a statistically significant savings of about S$784 (S$589-S$966) between patients treated in the EDOU compared to patients treated in the inpatient wards. 

**Table 4:** Average actual length of stay and cost outcomes ^ a^ Based on the actual length of stay in days (inclusive of stay in our hospital psychiatric facility) S$ - Singapore dollars

Outcomes	"Ward" group (n = 78)	EDOU group (n = 39)
Average length of stay (days)^a^	3.2 (2.3-4.4)	2.0 (1.2-3.4)
Average cost (S$)	2,637 (2,201-3,163)	1,300 (860-1,992)

Table 5 details the average costs stratified according to outcomes in both "ward" group and EDOU group respectively. Of note, when a patient is transferred to an external psychiatric facility, the cost of stay in the external psychiatric facility is not computed. Patients who required hospital stay under our hospital psychiatric facility tended to incur increased costs. 

**Table 5:** Average cost by outcomes for all inpatient ward and EDOU group patients S$ - Singapore dollars

Average cost by outcomes					
All inpatient ward group (n=78)		Median (S$)	Mean (S$)	Smallest bill (S$)	Largest bill (S$)
Discharged	65	1,843	2124	745	5,644
Discharged against advice	3	2,805	3023	2,441	3,823
Transferred to hospital psychiatric facility	6	6,230	7876	2,314	17,474
Transferred to external psychiatry facility	4	2,745	2837	2,543	3,314
EDOU group (n=39)		Median (S$)	Mean (S$)	Smallest bill (S$)	Largest bill (S$)
Discharged	23	707	730	551	986
Discharged against advice	2	NA	838	719	956
Transferred to inpatient medical ward	5	1,765	2,114	878	3,772
Transferred to hospital psychiatry facility	3	2,696	5,575	1,835	12,196
Transferred to external psychiatry facility	6	673	824	642	1,334

## Discussion

We found a "medical" LOS reduction of 31.9 hours or 57.8% and cost savings (S$784 per patient) without increased adverse effects when eligible patient groups are admitted to the EDOU instead of the inpatient wards.

To ensure that the "ward" and EDOU groups were comparable, we selected patients who required at least 21 hours of NAC and were suitable for admission to the EDOU that were instead admitted to the inpatient ward. We included patients from 2011 as historical controls to detect any differences in admitted inpatients characteristics prior to the start of EDOU from 2012. We did not find significant differences between the various groups. This assures us that the "ward" and EDOU groups were comparable for patient outcomes and allowed valid cost comparison. 

Compared to Tang’s study [7], the number of paracetamol-poisoned patients admitted to the hospital (ward and EDOU) was similar (77 and 76 per year respectively). The proportion of patients admitted to the EDOU showed a positive trend from 49% in 2013 to 75% in 2014 (70%). In both EDOU cohorts, the patients were young (median age <25), mostly female (more than 75%), had a median dose of 10g and had low rates of liver injury and hepatotoxicity. We also note that the majority of patients who ingested paracetamol did so for deliberate self-harm (81.3-100%), which is similar to the rates reported by another tertiary hospital [6]. This may be because our study population consisted mainly of those with single large ingestions (>95%).

Although our study cohort included patients where NAC was initiated more than eight hours after acute ingestion, those with large ingestions (dose >30g), as well as those with AT mildly elevated but <2x ULN at presentation, they did not affect the final results. These patients had an apparently higher risk of developing hepatotoxicity but they were distributed amongst both groups [16]. There were only four patients who developed liver injury and excluding them from the comparison did not affect the LOS estimate (Table 2 and 3). 

The "medical" LOS of patients in the "ward" group was 55.2 hours compared to the EDOU group at 23.3 hours, a significant finding on the background of rising healthcare costs and hospital bed shortage. A patient could save 31.9 hours (57.8% reduction) of admission if admitted to the EDOU instead of the ward. Tang et al. found a 65% reduction in LOS with an observation ward median LOS of 23 hours [7]. Beauchamp found LOS reduction of 27 hours for patients eligible for the observation ward but were instead admitted to the inpatient ward [10]. Offerman found that the LOS for patients with NAC was 67 hours [17], which is comparable to our patients with ward admission at 55.2 hours. The EDOU patient has the necessary interventions expedited due to the streamlining of medical care, psychosocial evaluation within the 23-hour stay. 

Despite the limitations of financial data abstraction where we were not able to quantify the cost incurred from inpatient hospital psychiatric facility stay separately, we found that EDOU admission resulted in a statistically significant savings of S$784 per patient. EDOUs have shown to result in LOS reduction and significant cost savings compared to inpatient care for a variety of medical conditions [9, 18]. Poisoning related studies have also shown cost savings when there was consultation with a toxicology service or poison center for poisoning. Isoardi et al. described a reduction in 1350 bed days and $2.25 million dollars over a period of two years at a tertiary hospital in Australia, following the establishment of a dedicated clinical toxicology unit [19]. Friedman et al. found that assistance by the poison center in Illinois reduced LOS by 0.6 days on average and potential savings of $2078 dollars per 10 patients [20]. Although the cost savings and LOS shortening in our study cannot be generalized to all poisonings, it shows that cost savings and reduced LOS can be achieved when patients are admitted to an EDOU with support from a toxicology service. Using our study cohort, we have an average of 39 patients a year (117 for three years). From this average, we extrapolate an annual savings of S$30576 for our hospital, from a savings of S$784 per patient.

The retrospective nature of this study exposes it to the biases of missing data and inaccurate coding. Our sample size is relatively small which limits the strength of our study results. Bias may have been introduced as one investigator was used to extract the data. Factors influencing the decision for EDOU or ward admission may not have been clearly documented, concealing possible confounders. We did not follow-up patient outcome after discharge although a previous study noted that delayed adverse poisoning effects were negligible [11]. Lastly, our single-centre study limits the generalization of our results to other centres that do not have a similar set up. 

## Conclusions

Our study suggests that selected patients with paracetamol poisoning that require a standard course of NAC can be safely and effectively managed in an EDOU supported by a toxicology service. There is cost savings and decreased LOS without an increase in adverse effects. Further studies are needed to verify our results. 
